# Effect of cognitive behavioral therapy for heart failure: A systematic review protocol of randomized controlled trial

**DOI:** 10.1097/MD.0000000000016803

**Published:** 2019-08-16

**Authors:** Wei-Qin Gao, Feng-Zhi Wang, Shu-Nan Wang, Feng-Na Zhang

**Affiliations:** a First Ward of Cardiology Department; b Department of Pharmacy, First Affiliated Hospital of Jiamusi University, Jiamusi; c First Ward of Cardiology Department, Qiqihar Medical College Affiliated No.3 Hospital, Qiqihar, China.

**Keywords:** cognitive behavioral therapy, effect, heart failure, randomized controlled trial, systematic review

## Abstract

**Background:**

This proposed study will systematically assess the effect and safety of cognitive-behavioral therapy (CBT) for heart failure (HF).

**Methods:**

We will search the following electronic databases for randomized controlled trials assessing the effect of CBT in patients with HF: PUBMED, EMBASE, Cochrane Library, Web of Science, Scopus, Chinese Biomedical Literature Database, China National Knowledge Infrastructure, VIP Information, and Wanfang Data from their inceptions to present without any language limitations. Two authors will independently conduct the study selection, data extraction, and methodological quality assessment. The methodological quality will be evaluated by Cochrane risk of bias tool.

**Results:**

This study will assess the efficacy and safety of CBT for patients with HF. The primary outcomes consist of depression and anxiety. The secondary outcomes comprise of all-cause mortality, change in body weight, urine output, change in serum sodium; and any adverse events.

**Conclusion:**

The results of this study will summarize the up-to-date evidence on the effect and safety of CBT for HF.

**PROSPERO registration number:**

PROSPERO CRD42019135932.

## 1 Introduction

Heart failure (HF) is a very common cardiovascular disease, and also a leading cause of morbidity and mortality worldwide.^[1,2]^ It has been reported that its prevalence is 1% to 2% among general population will be diagnosed with HF each year.^[3,4]^ Additionally, patients with HF also experience depression, anxiety, and poor health-related quality of life as common co-morbidities.^[5,6,7,8,9]^ Those complications have negative impacts on daily life, hospitalizations, and mortality rates in patients with HF.^[7,10]^ Therefore, it is very important to treat these complications effectively and timely.

Although pharmacological treatments are reported to treat these complications effectively, patients often experience complex medications and have more multiple co-morbidities.^[11,12,13]^ Non-pharmacological therapy, such as cognitive-behavioral therapy (CBT), has been demonstrated as potential treatments for depression, anxiety, as well as the health-related quality of life.^[14,15,16,17,18,19]^ However, these effects are not well systematically evaluated in patients with HF.

Theoretically, CBT may provide essential advantages over medication, including fewer adverse events, and more involvement of patients in their self-care. Several clinical studies have reported that CBT can effectively treat HF.^[16,20,21,22]^ However, to our best knowledge, no systematic review of randomized controlled trials (RCTs) has addressed the effects and safety of CBT in patients with HF. This study aims to determine the effect of CBT in patients with HF.

## 2 Methods

### 2.1 Inclusion criteria for study selection

#### 2.1.1 Study types

All RCTs that have assessed all types of CBT in patients with HF will be included without any restrictions of region and language. However, non-clinical studies, and non-RCTs will not be included.

#### 2.1.2 Participant types

All participants with HF will be included regardless the race, gender, and age.

#### 2.1.3 Intervention types

In the experimental group, patients must receive CBT.

In the control group, patients can receive any treatments, except CBT.

#### 2.1.4 Outcome types

Primary outcomes consist of depression, as measured by relevant scales, such as Hamilton Depression Rating Scale; and anxiety, as assessed by associated scales, such as Hamilton Anxiety Rating Scale. Secondary outcomes include all-cause mortality, change in body weight, urine output, change in serum sodium; and incidence of all adverse events.

### 2.2 Literature searches

The following databases will be searched from the inceptions to present without any language limitations: PUBMED, EMBASE, Cochrane Library, Web of Science, Scopus, Chinese Biomedical Literature Database, China National Knowledge Infrastructure, VIP Information, and Wanfang Data. All trials assessing the effect of CBT for patients with HF will be fully considered. The combination of the following search terms will be utilized to identify any potential eligible RCTs: “heart failure, congestive heart failure, cardiac failure, left ventricular failure, depressive disorder, depression, anxiety, quality of life, randomized controlled trial, clinical trial, controlled study and random”. The detailed sample search strategy for Cochrane Library is presented in Table 1. Similar detailed search strategies will also be applied to the other electronic databases. Additionally, we will also perform manual search of the reference lists of the included studies and relevant reviews.

**Table 1:**
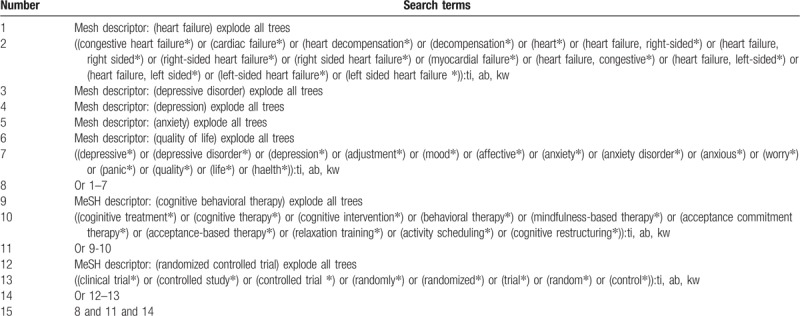
Search strategy applied in Cochrane Library database.

### 2.3 Study selection

**Figure 1:**
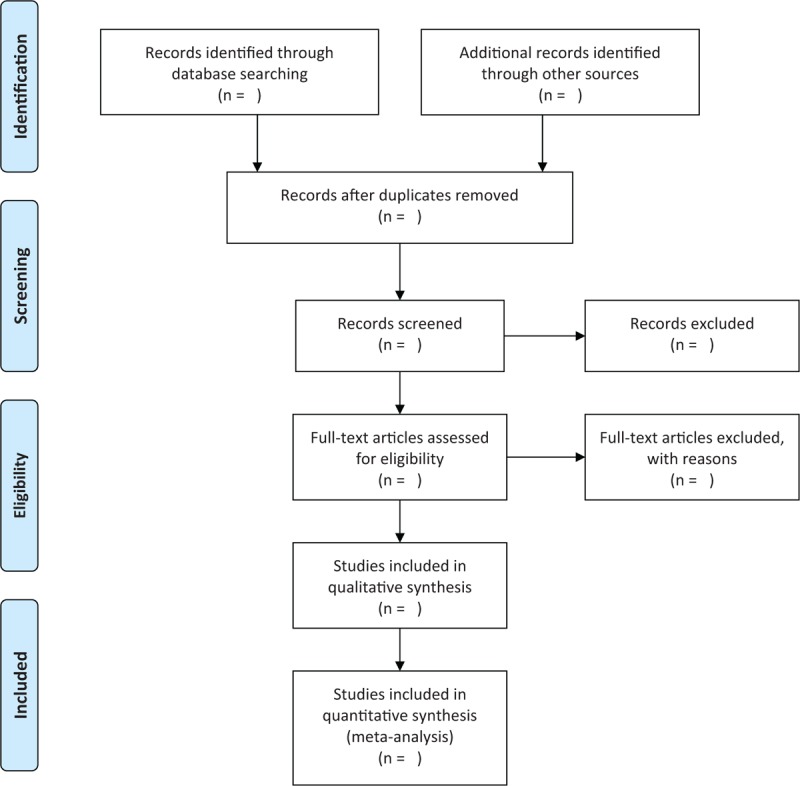
Flowchart of study selection.

Two authors will independently select the studies according to the predefined eligibility criteria. Any disagreements regarding the study selection will be solved by a third author through discussion. The titles and abstracts of all searched records will be read initially. After that, the full-texts of the rest studies will be further assessed if they can meet all eligible inclusion criteria. The study selection process is shown in Figure 1.

### 2.4 Data extraction and management

Two authors will independently perform data extraction by using a standardized data sheet. Any disagreements regarding the data extraction will be solved by a third author through discussion. The extracted data mainly comprise of title, author, and publication year, details of study, study methods, treatment details, and outcome measurements.

### 2.5 Methodological quality assessment

Cochrane Risk of Bias Tool will be used to assess the methodological quality for each included RCT by 2 independent authors. It consists of 7 items, and quality of each item will be evaluated using standard criteria of Cochrane Handbook for Systematic Reviews of Interventions. Any divisions will be settled down by consulting a third author.

### 2.6 Statistical analysis

ReMan 5.3 software will be used to assess the methodological quality, to pool the data and to carry out the meta-analysis. Continuous data will be presented as mean difference or standardized mean difference with 95% confidence intervals (CIs), while the dichotomous data will be shown as risk ratio with 95% CIs.

Heterogeneity will be identified using *I* ^*2*^ test. If value of *I* ^*2*^ is less than 50%, the acceptable heterogeneity is considered, and a fixed-effect model will be used to pool and analyze the data. Otherwise, the significant heterogeneity is considered, and a random-effect model will utilized to pool and analyze the data. If significant heterogeneity is detected, subgroup analysis will be carried out to identify the possible reasons that may cause high heterogeneity. If the heterogeneity is still significant after the subgroup analysis, data will not be pooled, and a narrative summary will be reported only. Moreover, sensitivity analysis will also be considered to perform to check the robustness of pooled results by removing low-quality trials. The value of *P* <.05 is defined as having statistical significance.

## 3 Discussion

CBT is widely applied to enhance depression and anxiety in patients with HF. However, the effect of CBT is still not certain. To our best knowledge, no systematic review has been explored to effect and safety of CBT for patients with HF. Therefore, it is very necessary to carry out a systematic review to assess the effect of CBT in patients with HF. This study will be helpful to physicians for the treatment of patients with HF and provide some useful information for clinical practice.

## Author contributions

**Conceptualization:** Wei-Qin Gao, Feng-Zhi Wang, Shu-Nan Wang, Feng-Na Zhang.

**Data curation:** Wei-Qin Gao, Feng-Zhi Wang, Shu-Nan Wang, Feng-Na Zhang.

**Formal analysis:** Wei-Qin Gao, Feng-Na Zhang.

**Funding acquisition:** Feng-Zhi Wang.

**Investigation:** Feng-Na Zhang.

**Methodology:** Feng-Zhi Wang, Feng-Na Zhang.

**Project administration:** Feng-Zhi Wang.

**Resources:** Wei-Qin Gao, Feng-Na Zhang.

**Software:** Wei-Qin Gao, Feng-Na Zhang.

**Supervision:** Feng-Zhi Wang.

**Validation:** Wei-Qin Gao, Feng-Zhi Wang, Shu-Nan Wang.

**Visualization:** Shu-Nan Wang, Feng-Na Zhang.

**Writing – original draft:** Wei-Qin Gao, Feng-Zhi Wang, Shu-Nan Wang, Feng-Na Zhang.

**Writing – Review & Editing:** Wei-Qin Gao, Feng-Zhi Wang, Shu-Nan Wang, Feng-Na Zhang.
